# CRISPR/Cas9-Based Editing of *Streptomyces* for Discovery, Characterization, and Production of Natural Products

**DOI:** 10.3389/fmicb.2018.01660

**Published:** 2018-07-24

**Authors:** Weixin Tao, Anna Yang, Zixin Deng, Yuhui Sun

**Affiliations:** Key Laboratory of Combinatorial Biosynthesis and Drug Discovery (Ministry of Education), Wuhan University School of Pharmaceutical Sciences, Wuhan, China

**Keywords:** natural product, *Streptomyces*, biosynthetic gene cluster, genome editing, CRISPR/Cas9

## Abstract

Microbial natural products (NPs) especially of the *Streptomyces* genus have been regarded as an unparalleled resource for pharmaceutical drugs discovery. Moreover, recent progress in sequencing technologies and computational resources further reinforces to identify numerous NP biosynthetic gene clusters (BGCs) from the genomes of *Streptomyces*. However, the majority of these BGCs are silent or poorly expressed in native strains and remain to be activated and investigated, which relies heavily on efficient genome editing approaches. Accordingly, numerous strategies are developed, especially, the most recently developed, namely, clustered regularly interspaced short palindromic repeats (CRISPR)/CRISPR associated (Cas) system reveals remarkable higher accuracy and efficiency for genome editing in various model organisms including the *Streptomyces*. In this mini review, we highlight the application of CRISPR/Cas9-based approaches in *Streptomyces*, focus on the editing of BGCs either *in vivo* or *in vitro*, as well as target cloning of large-sized BGCs and heterologous expression in a genetically manipulatable host, for discovery, characterization, reengineering, and production of potential pharmaceutical drugs.

## Introduction

*Streptomyces* species are known for the most prolific antibiotic producers and have provided a large number of clinical drugs during past decades. However, discovery of natural product (NP) drugs from these talented bacteria has suffered a blow after the Golden Age of NP discovery in 1950s–1960s, that is severely influenced by high-throughput screening of synthetic libraries and the low efficiency of traditional top-down screening strategies (Li and Vederas, 2009). Recently, great advances in next-generation sequencing technologies and computational resources reacquaint microbial genomes and are regarded as a huge reservoir of untapped NP biosynthetic gene clusters (BGCs; Rutledge and Challis, 2015; Weber and Kim, 2016; Kim et al., 2017); moreover, a vast majority of uncultured microorganisms in environments provide limitless possibilities for NP drugs discovery (Banik and Brady, 2010; Katz et al., 2016). For *Streptomyces*, the most gifted bacteria are supposed to possess 20–50 BGCs in a single genome, that greatly exceed the identified compounds (Challis, 2014; Baltz, 2017). Nevertheless, most of BGCs are silent or poorly expressed in native hosts under conventional laboratory culture conditions. To activate these cryptic BGCs, high-efficient approaches for genome editing and BGC engineering garner widespread attention and become a rapidly advancing field for NP drugs discovery (Hsu et al., 2014; Rutledge and Challis, 2015; Choi and Lee, 2016; Jakociunas et al., 2016; Li et al., 2017a; Ren et al., 2017; Zou et al., 2018).

Compared with other model organisms, like *Escherichia coli* and *Saccharomyces cerevisiae*, *Streptomyces* strains show poverty in genetic manipulation and most are recalcitrant for genome editing. In *Streptomyces*, recombinase-mediated homologous recombination has been commonly used for genome editing; however, the related protocols are often laborious and time-consuming (Gust et al., 2003; Komatsu et al., 2010; Fernandez-Martinez and Bibb, 2014; Li et al., 2017a). Until recently, application of clustered regularly interspaced short palindromic repeats (CRISPR)/CRISPR associated (Cas) system, especially the CRISPR/Cas9 system, has greatly facilitated high-efficiency genome editing (Jinek et al., 2012; Choi and Lee, 2016). Likewise, CRISPR/Cas9-based genome editing approaches have greatly accelerated insights into *Streptomyces* derived NP drugs. In this mini review, we summarize the recent developments and challenges of CRISPR/Cas9-based approaches for editing BGCs of *Streptomyces*; moreover, cloning and assembly of intact BGCs for heterologous expression are also emphasized.

## Crispr/Cas9 Advances the Genome Editing

CRISPR/Cas system functions as adaptive immune system in numerous bacteria and archaea, of which RNAs harboring “spacer” sequence from previously exposed bacteriophages help Cas proteins recognize and cleave the specific exogenous DNA (Barrangou et al., 2007; Grissa et al., 2007; Horvath and Barrangou, 2010). Since CRISPR/Cas system exhibits higher specificity and accuracy on sequence targeting, it has become excellent choice for precision genome editing (Jinek et al., 2012). CRISPR/Cas9, a type II CRISPR/Cas system, originally employs CRISPR RNA (crRNA) and *trans*-activating crRNA (tracrRNA) to form crRNA-tracrRNA duplex and then assists Cas9 nuclease to recognize and cleave target DNA harboring trinucleotide protospacer adjacent motif (PAM) and a 5’ end of 20 nucleotides complementary to the spacers (Deltcheva et al., 2011; Jinek et al., 2012; Hsu et al., 2014; Nishimasu et al., 2014). System reprogramming that fuses crRNA and tracrRNA into a synthetic single guide RNA (sgRNA) greatly facilitates preparation of transcripts and significantly promotes the application of CRISPR/Cas9 system (Jinek et al., 2012; Hsu et al., 2014). Reprogrammed CRISPR/Cas9 system has since been successfully used in a variety of organisms, including *S. cerevisiae* (DiCarlo et al., 2013), *Drosophila melanogaster* (Gratz et al., 2013), *Caenorhabditis elegans* (Friedland et al., 2013), plants (Jiang et al., 2013), and human embryos (Baltimore et al., 2015).

## *In Vivo* Strategies for Genome Editing in *Streptomyces*

*Streptomyces* are of utmost importance for novel NP drugs discovery, of which the investigating process relies heavily on high-efficiency genome editing. In *Streptomyces*, classic genome editing commonly achieves through homologous recombination with a suicide or temperature-sensitive or self-replicative plasmid, and requires intensive and time-consuming screening process. The application of CRISPR/Cas9 system for genome editing in *Streptomyces* started in 2015, and since then related approaches have been tremendously developed. As shown in **Table 1**, diversified approaches are widely used to edit or refactor BGCs for NP drugs discovery and characterization.

**Table 1:** Application of CRISPR/Cas9 strategies for genome/BGC editing in *Streptomyces* and some rare actinomycetes.

	Methods	BGCs	Function	Host (original/surrogate)	References
*In vivo* editing strategy	pCRISPomyces system	Phosphinothricin tripeptide	Deletion	*S. viridochromogenes*	Cobb et al., 2015
		Macrolactam, Lanthipeptide	Deletion	*S. albus*	
		Red, Actinorhodin (ACT)	Deletion	*S. lividans*	Cobb et al., 2015; Wang et al., 2016
		Eumelanin	Deletion	*Actinoplanes* sp. SE50/110	Wolf et al., 2016
		Formicamycins	Deletion	*S. formicae*	Qin et al., 2017
		Oxytetracycline	Site mutation/Deletion	*S. rimosus*	Jia et al., 2017
	pKCcas9dO system	ACT, Red, Ca2 + -dependent antibiotic (CDA)	Deletion/Site mutation	*S. coelicolor*	Huang et al., 2015
		BGC13	Replacement	*S. pristinaespiralis*	Li et al., 2017b
		Cryptic type I polyketide, Red, CDA	Replacement	*S. coelicolor*	
	pCRISPR-Cas9 system	ACT	Deletion	*S. coelicolor*	Tong et al., 2015
		Sceliphrolactam	Deletion	*Streptomyces* sp. SD85	Low et al., 2018
		Dynemicin	Deletion	*Micromonospora chersina*	Cohen and Townsend, 2018
	pCRISPR-dCas9 system	ACT	Reversible regulation	*S. coelicolor*	Tong et al., 2015
	CRISPR/Cas9-CodA(sm) combined system	ACT	Deletion	*S. coelicolor*	Zeng et al., 2015
	CRISPR–Cas9 knock-in strategy	Indigoidine	Promoter insertion	*S. albus*	Zhang et al., 2017
		ACT, Red	Promoter insertion	*S. lividans*	
		Alteramide A, Polycyclic tetramate macrolactam, FR-900098, type I polyketides	Promoter insertion	*S. roseosporus*	
		type III polyketide	Promoter insertion	*S. venezuelae*	
		Pentangular type II polyketide	Promoter insertion	*S. viridochromogenes*	
Direct cloning	CATCH	Jadomycin	Cloning	*S. venezuelae*	Jiang et al., 2015
		Chlortetracycline	Cloning	*S. aureofaciens*	
BGC refactoring in yeast	mCRISTAR	Tetarimycin, Lazarimide, AB1210	Promoter refactoring	*S. albus*	Kang et al., 2016
*In vitro* editing strategy	ICE	RK-682	Deletion/Insertion	*S. lividans*	Liu et al., 2015
		Holomycin	Deletion	*S. albus*	
		Tü 3010	Deletion	*S. avermitilis*	Tao et al., 2016
	CRISPR/Cas9 system combined with Gibson assembly	Pristinamycin II	Vector refactoring	*S. pristinaespiralis*	Li et al., 2017b
		Chloramphenicol	Vector refactoring	*S. coelicolor*	


**FIGURE 1:**
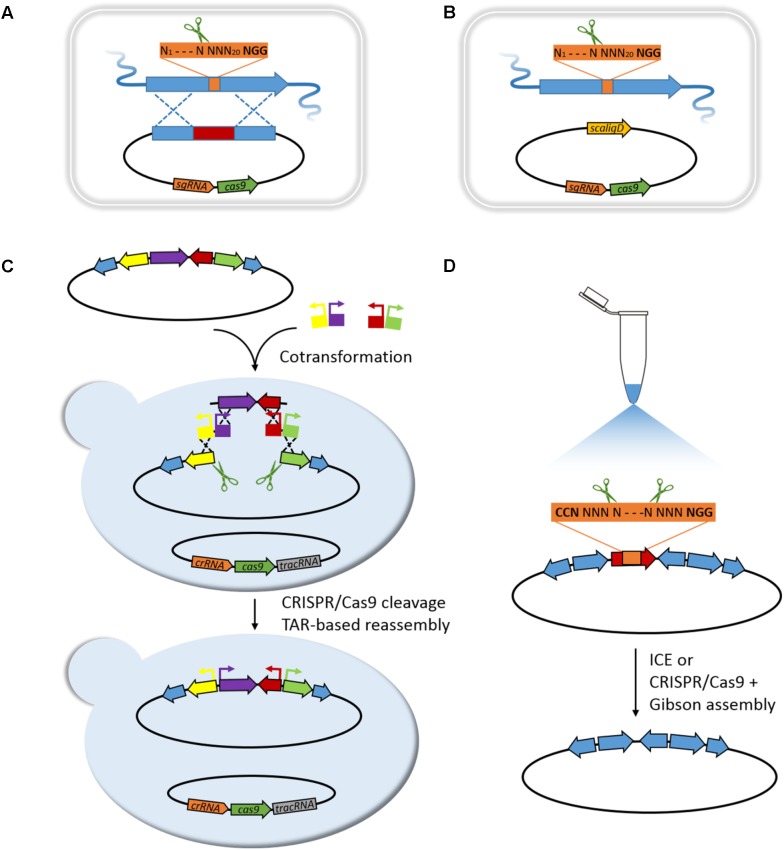
Editing of BGCs based on CRISPR/Cas9 strategies. **(A)** HDR-mediated editing in *Streptomyces*. Gene deletion, point mutation, or promoter substitution can be performed, respectively, when the repairing template carries corresponding deletion, point mutation, or promoter replacement. Multiplex loci editing can be achieved by equipping multiplex sgRNA cassettes and corresponding HRTs. **(B)** NHEJ-mediated editing in *Streptomyces*. Small sized deletion, insertion, or substitution (mostly 1–3 bp) close to the target site can be achieved by using a reconstituted NHEJ system that co-expressing the *scaligD* in *S. coelicolor*. **(C)** mCRISTAR for BGC refactoring in yeast. **(D)** *In vitro* editing of BGC by ICE or SRISPR/Cas9 coupling Gibson assembly.

Cobb et al. (2015) first introduced CRISPR/Cas9 system for genome editing in *Streptomyces.* The pCRISPomyces-2 system equips a codon-modified Cas9 nuclease driven by a strong promoter, a sgRNA expression cassette, and a 2 kb homology repair template (HRT). It first specifically generates a double-strand break (DSB) at target site by Cas9 nuclease under the guidance of sgRNA harboring a custom-designed spacer, and then repairs the resulting chromosome break by homology-dependent repair (HDR) system in the presence of HRT and introduces chromosomal deletions ranging from 20 bp to 31 kb with an efficiency ranging from 70 to 100% (**Figure 1A**). Multiplex genome editing may be achieved by equipping multiplex sgRNA cassettes and corresponding repairing templates in a pCRISPomyces system, and excision of 31 kb BGC of undecylprodigiosin (Red) in *Streptomyces lividans* 66 has thus successfully obtained.

Slightly afterward, three different groups successively applied CRISPR/Cas9 system in *Streptomyces* for diverse applications. The pKCcas9dO system by Lu group similarly revealed high editing efficiency of CRISPR/Cas9 system in *Streptomyces coelicolor* M145 for single gene/BGC deletion, as well as multiplex genes/BGCs deletions (Huang et al., 2015). Besides, a point mutation editing strategy that CRISPR/Cas9 cleaves chromosome DNA at specific site guided by synthetic sgRNA, and then the HDR in *S. coelicolor* helps repair DSB in assistance of HRT with designed point mutation (AAG of 262–264 nucleotides in *rpsL* was changed to GAA), has performed to convert Lys88 to Glu in *rpsL* (**Figure 1A**). Tong et al. (2015) have thoroughly investigated editing efficiency when repairing Cas9-generated site-specific DSBs by non-homologous end joining (NHEJ) system in *S. coelicolor* A3(2). It revealed an incomplete NHEJ system in *S. coelicolor* that lacking a core component LigD, and led to randomly sized deletions around target site. Reconstitution of this defective NHEJ system by complementing *Streptomyces carneus* derived ScaligD has increased editing efficiency up to 77% and qualified the mutations to 1–3 bp deletion/insertion/substitution in most cases (**Figure 1B**). Moreover, high precision genome editing efficiency near 100% achieved when supplying the HRT. In the study, CRISPRi using a catalytically inactive Cas9 nuclease (dCas9) has also been developed, to target promoter region or open reading frame of *actIORF1* for reversible regulation of actinorhodin production in *S. colicolor*. In the same year, Sun group developed an extraordinary CRISPR/Cas9-CodA(sm) combined system, using CodA(sm), the D314A mutant of cytosine deaminase to convert 5-fluorocytosine to toxic 5-fluorouracil, as an efficient counter-selection approach to select for progenies lost recombinant plasmid, which greatly accelerates screening process (Zeng et al., 2015). Besides, a most significant feature that differs from above three systems is application of a segregationally unstable *sti^-^* pIJ101-derived shuttle vector. The behavior of self-replication with high copy number of about 50 per chromosome of this deliver vector can produce large amounts of single-strand plasmid DNA and plenty of template DNAs, resulting in dramatically high efficiency of double cross-over recombination and frequency of target mutant (Zeng et al., 2015). All the above reports facilitate rapid progress for genome editing in *Streptomyces*, since CRISPR/Cas9 helps select against wild-type sequence in the presence of HRTs. CRISPR/Cas9 system also enables activation of cryptic BGCs in *Streptomyces*. Zhao group utilized CRISPR/Cas9-mediated knock-in strategy for efficient and precise insertion of constitutive promoters upstream of main biosynthetic operons or pathway-specific activators, and triggered production of novel NPs of different classes in multiple *Streptomyces* species (**Figure 1A**; Zhang et al., 2017).

To date, CRISPR/Cas9 system has been applied for genome editing in *Streptomyces* for 3 years, the high specificity and efficiency made it the most attractive technology in that field. Its application has now extended to many non-model *Streptomyces* strains, like *Streptomyces formicae* from the African fungus-growing plant-ant *Tetraponera penzigi* (Qin et al., 2017), *Streptomyces rimosus* with distinctive chromosome terminal and core regions (Jia et al., 2017), *Streptomyces* sp. SD85 from tropical mangrove sediments (Low et al., 2018), and some rare actinomycetes like *Actinoplanes* sp.SE50/110 (Wolf et al., 2016) and *Micromonospora chersina* (Cohen and Townsend, 2018). However, *in vivo* application of this fascinating technology in *Streptomyces* is confined to the strains are genetically tractable, missing out on a vast amount of precious BGC resources from genetically intractable strains or yet uncultured strains. In that case, acquiring and refactoring of intact BGCs for heterologous expression in a genetically tractable surrogate host could be alternatively considered.

## Crispr/Cas9-Mediated Bgcs Cloning and Refactoring for Heterologous Expression

### Cloning of Large-Sized BGCs

A variety of cryptic BGC awakening approaches, like pathway-specific/global regulator manipulation, promoter refactoring, and ribosome engineering, have been used for NPs discovery in *Streptomyces*. However, most require genetic manipulation of native strains thus are constrained in genetically intractable strains or BGCs from environmental DNA (eDNA; Rutledge and Challis, 2015; Weber et al., 2015; Zhang et al., 2016). Strategies for heterologous expression of BGCs in a genetically manipulatable host can perfectly circumvent above bottleneck, but cloning and editing of large-sized BGCs (sometimes over 100 kb) remain challenging. For cloning large-sized DNAs, classic strategies generally utilize randomly digested genomic libraries; however, the screening process is always laborious and it is arduous for packaging intact BGCs over 100 kb in a single vector. Previous precision cloning strategies often utilize restriction enzymes (REs) to release target BGCs that subsequently acquired by coupling diverse DNA capturing strategies. Linear–linear homologous recombination (LLHR) uses RecE/T mediated homologous recombination for direct capture of REs generated genome segments and is widely used for direct cloning of NP BGCs from *Streptomyces* (Fu et al., 2012; Nah et al., 2017). Gibson assembly coupling REs cleavage is also used for capturing BGCs, and accordingly, the conglobatin cluster has target cloned by Leadlay group (Zhou et al., 2015). Transformation-associated recombination (TAR) cloning uses homologous recombination in *S. cerevisiae* to capture REs generated BGC segments, and has employed for cloning BGCs of taromycin A (Yamanaka et al., 2014), alterochromide (Ross et al., 2015), and thiotetronates (Tang et al., 2015). However, these REs-dependent approaches are severely constrained for broader application since appropriate RE cutting sites do not regularly exist close to BGC terminals. CRISPR/Cas9 system perfectly addresses the limitation, that target cleaves the DNA guided by a synthetic sgRNA, allowing target cloning of large-sized BGCs. Wang et al. (2015) tentatively applied CRISPR/Cas9 system as REs *in vitro* to linearize a large vector (22 kb) and subsequently seamlessly assembled with a small DNA using Gibson assembly (Wang et al., 2015). For precision acquiring large-sized DNAs harboring NP BGCs, Jiang et al. (2015) developed Cas9-assisted targeting of chromosome segments (CATCH), which allows target cloning of intact BGCs up to 100 kb that cleaved by CRISPR/Cas9 at specific sites guided by custom-designed sgRNAs and subsequent target captured by Gibson assembly (Jiang et al., 2015). Simultaneously, Lee et al. (2015) combined CRISPR/Cas9 with TAR cloning that employs homologous recombination in yeast to target capture CRISPR/Cas9 released chromosomal segments and dramatically accelerated capture efficiency of TAR cloning up to 32% (Lee et al., 2015). Soon after, CRISPR/Cas9 system coupling TAR cloning was further applied to construct even megabase-sized DNA segments. Zhou et al. (2016) developed Cas9-facilitated homologous recombination assembly (CasHRA), which co-introduces large circular DNAs into *S. cerevisiae* and release the target DNA segments by CRISPR/Cas9 for subsequent assembly by homologous recombination. It provides an alternative for assembly of large-sized BGCs over 100 kb, using DNAs obtained from cosmid libraries of *Streptomyces* or eDNA. However, it involves assembly steps and tends to be time-consuming.

### CRISPR/Cas9-Mediated Editing of BGCs

Acquiring of intact BGCs of interest is the first step to heterologously investigate the novel NP drugs. Editing of acquired BGCs is generally required for successful heterologous expression. Routine strategies for BGCs editing are always constrained by difficulty of handling large-sized DNAs, and are always laborious. λ-Red recombination mediated PCR-targeting has often used for editing BGC by creating gene replacements/deletions; however, residues like antibiotic selection markers or FRT sequence remain at editing sites, and unintended recombination may raise from repetitive sequences of such modular PKS or NRPS genes (Gust et al., 2003; Yamanaka et al., 2014). λ-Red recombination also enables promoter refactoring or domains/modules exchange for characterization of NPs biosynthesis (Nguyen et al., 2006; Du et al., 2013). Recently, a more facile promoter refactoring approach based on homologous recombination in *S. cerevisiae* has been developed by Brady group, that enables multiplex promoter refactoring in a single TAR reaction (Montiel et al., 2015). Based on this, production of eDNA-derived indolotryptoline antiproliferative agents, lazarimides A and B, was activated. Nevertheless, the refactoring rate is relatively low. Homologous recombination in yeast could be greatly improved if specific DSBs are introduced at recombination sites (Storici et al., 2003; Storici and Resnick, 2006; Lee et al., 2015). Accordingly, Brady group developed multiplexed-CRISPR-TAR (mCRISTAR) approach, which introduces CRISPR/Cas9 system to specifically create DSBs across target recombination sites (Kang et al., 2016). With mCRISTAR, multiplex CRISPR/Cas9 generated operon fragments can be reassembled with synthetic promoter cassettes by homologous recombination, and are capable of achieving four promoters exchange simultaneously in a single round using one auxotrophic marker selection, with efficiency up to 80% (**Figure 1C**). General applicability of mCRISTAR has been validated by applying to activate three different cryptic BGCs coding for tetarimycin, lazarimide, and AB1210, indicating a powerful and promising technology for discovery of novel NP drugs from cryptic BGCs resource.

In contrast to the above *in vivo* strategies for BGC editing based on homologous recombination in *E. coli* or *S. cerevisiae*, Sun group developed a new *in vitro* CRISPR/Cas9-mediated editing (ICE) system for high-efficient BGCs editing (Liu et al., 2015). ICE system allows a complete *in vitro* operating process with normal molecular operations, which cleaves BGCs at specific sites guided by synthetic sgRNAs and ligates the blunt ends that are repaired by T4 polymerase, to create gene in-frame deletion/replacement/insertion mutations (**Figure 1D**). With ICE system, BGCs of tetronate RK-682 and dithiolopyrrolone holomycin were readily edited (Liu et al., 2015), especially for Tü 3010, a particular thiotetronate antibiotic, various gene in-frame deletions were rapidly constructed and accordingly deciphered biosynthesis of this exceptional thiotetronate structure (Tao et al., 2016). Soon afterward Lu group utilized a similar *in vitro* approach that coupling CRISPR/Cas9 system with Gibson assembly to refactor the bacterial artificial chromosome vector harboring BGC of pristinamycin II for following multiplexed site-specific genome engineering in *Streptomyces* (**Figure 1D**; Li et al., 2017b). The above two examples indicate that *in vitro* application of CRISPR/Cas9 could be of wide applicability for BGCs editing, especially coupling the subsequent heterologous expression of BGCs for NP drugs discovery, characterization, and engineering. Nevertheless, optimization of *in vitro* strategies for multiplex loci refactoring of BGCs is of great necessity, and coupling of CRISPR/Cas9 system with Gibson assembly may preliminarily address the problem.

## Conclusion

In conclusion, CRISPR/Cas9 system has proved to be a powerful technology for genome editing or BGC refactoring due to the outstanding features, like higher sequence specificity, artificial guided targeting, and high editing efficiency. Its applications of genome editing specialized for *Streptomyces* are still relatively narrower in range, especially for the strains little studied. Thus, more efficient and convenient CRISP/Cas tools are of urgent requirement. For instance, diversified CRISPR/Cas systems like Cpf1 (Zetsche et al., 2015; Fonfara et al., 2016; Li et al., 2016), the newly identified class 2 type V CRISPR/Cas protein, xCRISPR/Cas9 (Hu et al., 2018), the most recently evolved CRISPR/Cas9 system with broad PAM compatibility, and even the CRISPR/Cas systems from *Streptomyces* (Choi and Lee, 2016) could be introduced for diverse applications in *Streptomyces*, to advance the researches on NP drugs and open a new era for NP drugs discovery.

## Author Contributions

WT and YS wrote the manuscript. All authors revised and approved the manuscript.

## Conflict of Interest Statement

The authors declare that the research was conducted in the absence of any commercial or financial relationships that could be construed as a potential conflict of interest. The reviewer SC and handling Editor declared their shared affiliation.
